# Effects of embryo injected with ochratoxins A on hatching quality and jejunum antioxidant capacity of ducks at hatching

**DOI:** 10.3389/fvets.2022.944891

**Published:** 2022-08-09

**Authors:** Jinhui Liu, Xiayu Jiang, Xin Peng, Yihan Yuan, Yu Shen, Yongxia Li, Zixing Yan, Xi Yuan, Ye Yang, Shuangshuang Zhai

**Affiliations:** College of Animal Science, Yangtze University, Jingzhou, China

**Keywords:** ochratoxins A, duck, embryo injection, hatching quality, jejunal antioxidant capacity

## Abstract

Numerous studies have shown that ochratoxins A (OTA) exerts diverse toxicological effects, namely, hepatotoxicity, nephrotoxicity, genotoxicity, enterotoxicity, and immunotoxicity. The main objective of this study was to investigate the influence of embryonic exposure to OTA by different injection times and OTA doses on hatching quality and jejunal antioxidant capacity of ducks at hatching. In total, 480 fertilized eggs were weighed and randomly assigned into a 4 × 4 factorial design including four OTA doses (0, 2, 4, and 8 ng/g egg) on 8, 13, 18, and 23 of embryonic development (E8, E13, E18, and E23). Each treatment included 6 repeats with 5 eggs per repeat. The results showed that the injection time affected the hatching weight (*P* < 0.0001). The relative length of the jejunum and ileum on E18 and E23 was lower than on E8 and E13 (*P* < 0.05). Injection time, doses, and their interaction had no effect on jejunum morphology, namely, villous height (Vh), crypt depth (Cd), and villous height/crypt depth ratio Vh/Cd (*P* > 0.05). The injection time affected the activities of Superoxide dismutase (SOD) (*P* < 0.0001), total antioxidant capacity (T-AOC) (*P* < 0.05) and the malondialdehyde (MDA) content (*P* < 0.0001). The activity of SOD and T-AOC activities in the jejunum of ducklings injected with OTA at the E8 and E13 was lower than that injected at the E18 (*P* < 0.05). The highest MDA content was observed in ducklings injected with OTA at the E13 (*P* < 0.05). The injection time (*P* < 0.0001), OTA doses and their interaction affected the contents of IL-1β (*P* < 0.05), which significantly increased especially on E13. In conclusion, the embryo injected with ochratoxins A affected the hatching weight, the relative length of jejunum and ileum, decreased the antioxidant capacity and increased the content of proinflammatory cytokine IL-1β of the jejunum.

## Introduction

Mycotoxins are secondary metabolites produced by filamentous fungi that cause a toxic response when ingested by humans and animals. *Fusarium, Aspergillus*, and *Penicillium* are the most abundant molds that produce mycotoxins and contaminate human foods and animal feeds through fungal growth prior to and during harvest or improper storage (1). According to incomplete statistics, 30% of maize and 5% of soybean meals were positive for aflatoxins (2). The detection rates of aflatoxin B1, deoxynivalenol, zearalenone, and fumonisin in laying hens feed were 83.16, 93.68, 94.74, and 100%, respectively, and those in meat-type poultry feed were 89, 96, 85, and 94.74% in Southwest China (3). The presence of mycotoxins in poultry diets induced a reduction in body weight, egg production, and hatchability and increased susceptibility to disease mortality in chickens (4–6).

Ochratoxin A (OTA) is a mycotoxin that is produced by certain species of *Aspergillus* and *Penicillium* including *A. ochraceus, A. carbonarius*, and *P. Pverrucosum* (7). Many studies have shown that OTA exerts diverse toxicological effects, namely, hepatotoxicity, nephrotoxicity, genotoxicity, immunotoxicity, and enterotoxincity (8–10). A study showed that OTA in mixed feed and feedstuffs used in some laying hens was found to be positive in 64 of the 89 samples analyzed (11). Mycotoxins may remain in the tissues and eggs of poultry-fed moldy feed. Among 115 chicken meat and 80 egg samples, 41% of the meat samples, and 35% of the egg samples were positive for OTA (12).

The decrease in the quality of eggs such as, eggshell thickness and egg weight also indicated the effects of OTA in the different parts of the female reproductive system including shell glands (13). OTA causes teratogenic effects in the embryos in the form of anophthalmia, mandibular hypoplasia, maxillary retrognathism, everted viscera, microphthalmia, spina bifida, exencephaly, reduced body size, and appear immunosuppression (14–16).

The OTA exerts diverse toxicological effects, namely, hepatotoxicity, nephrotoxicity, genotoxicity, immunotoxicity, and enterotoxication, and affects embryo development. The young animals and ducks or turkeys are usually the most sensitive species with regard to their response to OTA (17). Embryonic ducks may be more sensitive to OTA, and there is a lack of research in this field. Therefore, the present study evaluated the effects of different doses and times of OTA injection on the hatching quality, jejunum morphology, antioxidant capacity, and cytokines of ducks during the embryonic stage.

## Materials and methods

### Eggs and birds

A total of 650 eggs, which were purchased from the HuBei Shendan Health Food Co. Ltd., were selected and unfertilized eggs were removed by light candling on day 5 of embryonic development, and to determine the air cell border. Eggs were individually weighed, and then incubated in an incubator (Keyu Co, China) at 37.5°C and 60% relative humidity, and turned every 90 min at a 90° angle during the first 24 days of incubation.

### Experimental design

In total, 480 fertilized eggs were individually weighed and randomly assigned into a 4 × 4 factorial design at four different injection times (E8, E13, E18, and E23) at four OTA doses [0, 2, 4, and 8 ng/g egg (Pribolab, Qingdao, China)]. The control group was injected with 0.1 ml NaHCO_3_ solution. OTA was dissolved in 0.1 ml of 0.1 M NaHCO_3_, which was sterilized by autoclaving. The required amount of OTA was placed onto the yolk sac, and the hole was sealed with melted wax. After dosing eggs were incubated at 37.8°C and 60% relative humidity.

### Embryo injection method

Embryos received treatment by direct injection into the yolk sac according to the standard techniques. The surface was wiped with 75% ethanol, and a small hole was drilled in the shell over the yolk. Care was taken not to tear the shell membrane. Injections were made at a 45° angle through the hole, and the solvents were injected into the yolk with a 1 ml sterile syringe. The holes were sealed with a small piece of tape.

### Sample procedures

In total, six birds per treatment were decapitated and parts of their jejunum were snap-frozen in liquid nitrogen and stored at −80°C until further analyses.

### Newly-hatched duck weight and organ index

In evaluating a newborn duck's physical quality, the analysis was conducted at the end of the hatching window. All the newly hatched ducks were individually weighed and six birds per treatment were decapitated for organ analyses. The weights of the organ, including heart, liver (without the gall bladder), spleen, pancreas, kidney, gizzard, proventriculus, and bursa of Fabricius were recorded, and their relative weights were calculated in relation to the live body weight (BW) of the birds. Besides, the weights and length of the duodenum, jejunum, ileum, cecum, and rectum were recorded, both their relative weights and their relative length were calculated in relation to the live BW of the birds.

### Histomorphometry

Segments were removed from the jejunum. The samples were flushed with physiological saline to remove all the contents and fixed in 4% paraformaldehyde. Three cross-sections were prepared for each sample after staining with hematoxylin and eosin using standard paraffin embedding procedures. The evaluated morphometric indices were as follows: villus height (Vh, from the tip of the villus to the crypt), crypt depth (Cd, from the base of the villus to the submucosa), and the villus height-to-crypt depth ratio (Vh/Cd). Morphological indices were measured using an image processing and analysis system (ImageJ).

### Antioxidant and inflammation of jejunal

To investigate the jejunum oxidation status of the hatched ducks, including superoxide dismutase (SOD), total antioxidant capacity (T-AOC), and malondialdehyde (MDA) and jejunum inflammatory cytokines, namely, interleukin 1β (IL-1β), tumor necrosis factor α (TNF-α), and interleukin (IL-6) were quantified using commercial diagnostic kits for jejunum analysis (Jiancheng Bioengineering Institute, Nanjing, China).

### Statistical analysis

All the data were analyzed by two-way ANOVA SAS software (SAS Institute Inc., Cary, NC). Data were further analyzed in a 4 × 4 (time × dose) factorial arrangement of treatments with a model that included the main effects of time and dose, as well as their interaction. Data are expressed as the means and standard error of the mean (SEM). Significant differences were set at *P* ≤ 0.05.

## Results

### Hatching weight and organ index

The effect of time and doses of an embryo injected OTA on hatching weight and organ index of ducks at hatching are presented in Table 1. Different OTA doses and the interaction between injection time and doses had no influence on the hatching weight and the relative weights of the heart, liver (without the gall bladder), spleen, pancreas, kidney, gizzard, proventriculus, and bursa of Fabricius (*P* > 0.05). Compared with E18 and E23, injected on E8 and E13 decreased the hatching weight (*P* < 0.05). Regardless of the OTA injection dose, the hatching weight of ducklings injected with OTA at the 8^th^ and 13^th^ embryonic age was lower than other injected ages (*P* < 0.0001). The largest hatching weight was obtained when the embryo age of OTA injection was 18^th^ and the concentration was 4 ng/g egg (*P* = 0.0016). The relative weight of the spleen on E8 was lower than that on E18 (*P* < 0.05).

**Table 1:** Effect of time and doses of an embryo injected OTA on hatching weight and organ index of ducks at hatching^1^ (g/kg).

		**Hatching weight (g)**	**Heart**	**Liver**	**Kidney**	**Spleen**	**Pancreas**	**Gizzard**	**Proventriculus**	**Bursa fabricius**
**Time**	**Dose**									
E8	0	38.112^abcd^	8.978	31.192	7.533	0.479	2.610	43.777	6.764	1.363
E8	2	37.362^bcd^	9.487	30.116	7.576	0.370	1.846	37.934	6.668	1.547
E8	4	40.883^ab^	9.575	30.058	6.752	0.462	1.707	41.375	7.326	1.448
E8	8	38.313^abcd^	9.143	28.129	6.057	0.529	1.947	42.851	6.986	1.270
E13	0	38.334^abcd^	9.496	30.068	7.159	0.677	1.873	38.977	6.255	1.570
E13	2	38.265^abcd^	8.505	31.216	7.446	0.481	1.827	46.112	7.633	1.378
E13	4	35.31^d^	8.749	29.023	6.876	0.459	2.012	44.012	7.251	1.236
E13	8	36.137^cd^	9.575	27.858	7.399	0.515	2.053	37.998	6.829	1.187
E18	0	41.89^a^	8.889	28.349	6.642	0.680	1.849	38.438	6.656	1.923
E18	2	40.504^ab^	8.864	29.678	7.171	0.689	1.790	37.388	6.941	1.420
E18	4	41.862^a^	8.842	30.490	6.676	0.618	1.913	39.512	6.777	1.578
E18	8	40.028^abc^	9.163	28.709	7.818	0.535	1.841	38.116	6.561	1.364
E23	0	41.552^a^	8.395	29.204	7.642	0.617	2.305	43.387	7.097	1.342
E23	2	41.36^ab^	9.499	30.193	6.980	0.511	1.631	40.123	6.582	1.482
E23	4	40.198^ab^	9.221	30.896	7.753	0.456	1.214	38.189	7.490	2.247
E23	8	40.246^ab^	9.662	31.417	8.392	0.509	1.750	43.166	7.538	2.142
SEM		1.111	0.604	1.481	0.669	0.0816	0.256	5.753	0.509	0.386
*P*-value		0.0016	0.966	0.9	0.754	0.203	0.439	0.333	0.868	0.859
Main effect										
Time										
	E8	38.668^b^	9.295	29.873	6.979	0.46^b^	2.027	41.484	6.936	1.407
	E13	37.012^b^	9.081	29.541	7.22	0.533^ab^	1.941	41.774	6.991	1.342
	E18	41.071^a^	8.939	29.306	7.077	0.63^a^	1.848	38.363	6.733	1.571
	E23	40.839^a^	9.194	30.427	7.691	0.523^ab^	1.725	41.216	7.176	1.803
SEM		0.583	0.32	0.763	0.344	0.042	0.132	1.326	2.262	0.199
Dose										
	0	39.972	8.939	29.703	7.244	0.613	2.159	41.144	6.693	1.549
	2	39.564	9.088	30.301	7.293	0.513	1.773	40.389	6.956	1.457
	4	39.564	9.097	30.117	7.014	0.498	1.711	40.772	7.211	1.627
	8	38.681	9.385	29.028	7.417	0.522	1.897	40.532	6.978	1.491
SEM		0.607	0.305	0.748	0.338	0.0412	0.129	1.299	0.257	0.195
*P-*value										
Time		<0.0001	0.834	0.718	0.413	0.034	0.695	0.161	0.645	0.413
Dose		0.446	0.806	0.642	0.818	0.183	0.100	0.993	0.574	0.945
Time*dose		0.308	0.867	0.808	0.639	0.734	0.458	0.246	0.807	0.756

SEM, Standard error of the mean.

^a−b−c^Values within a column with no common letters differ significantly (P < 0.05).

^1^Each value represents the mean of 6 replicate.

### Intestinal index and histomorphometry

Table 2 presents the effect of time and doses of an embryo injected OTA on the intestinal relative weight of ducks at hatching. The OTA injected time, doses, and their interaction had no effect on the intestinal relative weight of ducklings at hatching, namely, the relative weight of duodenum, jejunum, ileum, cecum and rectum (*P* > 0.05). Different OTA doses and the interaction between inject time and dose had no influence on intestinal relative length, namely, duodenum, jejunum, ileum, cecum, and rectum (*P* > 0.05, Table 3). A main effect of time was observed for the relative length of the jejunum (*P* = 0.012) and ileum (*P* = 0.013). Regardless of OTA injected doses, the relative length of jejunum and ileum of ducklings injected with OTA at 8^th^ and 18^th^ embryonic age was longer than other injected ages (*P* < 0.05). Injection time, OTA doses and their interaction had no effect on jejunum morphology, namely, villous height, crypt depth, and Vh/Cd (*P* > 0.05) (Table 4).

**Table 2:** Effect of time and doses of an embryo injected OTA on the intestinal relative weight of ducks at hatching^1^ (g/kg).

**Item**		**Duodenum**	**Jejunum**	**Ileum**	**Cecum**	**Rectum**
**Time**	**Dose**					
E8	0	7.822	13.355	11.935	1.653	5.424
E8	2	5.803	10.242	10.649	1.298	3.272
E8	4	5.671	11.777	12.286	1.921	3.918
E8	8	6.223	11.470	11.756	2.015	4.482
E13	0	5.929	10.974	11.082	1.352	5.072
E13	2	5.662	12.026	11.877	1.592	3.123
E13	4	5.997	11.477	11.197	1.942	3.319
E13	8	6.092	10.713	11.854	2.173	4.088
E18	0	5.733	10.602	11.066	1.762	3.181
E18	2	5.445	9.685	10.339	1.787	3.264
E18	4	5.027	9.711	10.413	0.811	3.029
E18	8	6.158	11.248	13.173	1.741	3.920
E23	0	6.353	11.457	12.533	1.783	3.731
E23	2	5.775	9.670	9.578	1.468	4.839
E23	4	4.443	9.065	9.369	1.419	2.590
E23	8	6.518	10.864	11.594	2.734	3.603
SEM		0.549	0.985	1.004	0.355	0.799
*P*-value		0.313	0.541	0.498	0.224	0.659
Main effect						
Time						
	E8	6.38	11.711	11.656	1.722	4.274
	E13	5.92	11.297	11.502	1.765	3.901
	E18	5.59	10.312	11.247	1.525	3.348
	E23	5.772	10.264	10.769	1.851	3.691
SEM		0.287	0.516	0.526	0.186	0.419
Dose						
	0	6.459	11.597	11.654	1.638	4.352
	2	5.671	10.406	10.611	1.536	3.624
	4	5.284	10.508	10.816	1.523	3.214
	8	6.248	11.074	12.094	2.165	4.023
SEM		0.277	0.497	0.507	0.179	0.404
*P*-value						
Time		0.537	0.250	0.862	0.639	0.587
Dose		0.059	0.444	0.15	0.076	0.343
Time*dose		0.576	0.641	0.515	0.315	0.652

SEM, Standard error of the mean.

^1^Each value represents the mean of 6 replicates.

**Table 3:** Effect of time and doses of an embryo injected OTA on the intestinal relative length of ducks at hatching^1^ (cm/kg).

**Item**		**Duodenum**	**Jejunum**	**Ileum**	**Cecum**	**Rectum**
**Time**	**Dose**					
E8	0	231.78	501.70	457.03	79.73	93.80
E8	2	221.04	486.01	464.81	93.93	84.06
E8	4	239.70	493.03	457.01	83.23	92.83
E8	8	222.94	508.49	485.03	96.56	98.68
E13	0	224.76	476.26	486.67	89.35	86.82
E13	2	241.21	517.62	469.08	101.87	91.50
E13	4	234.88	500.15	443.16	90.30	96.37
E13	8	256.03	456.58	482.11	97.61	99.60
E18	0	200.96	445.66	426.2	89.74	79.78
E18	2	214.72	432.41	413.40	88.45	82.44
E18	4	192.28	444.02	430.11	84.25	85.67
E18	8	241.72	480.13	445.78	92.80	92.71
E23	0	238.54	462.99	439.40	92.76	90.32
E23	2	226.76	438.41	415.84	88.84	92.97
E23	4	192.80	385.81	369.79	83.10	78.09
E23	8	233.74	461.97	429.3	94.38	89.23
SEM		16.105	27.173	26.546	7.08	8.78
*P*-value		0.332	0.162	0.2803	0.74	0.9268
Main effect						
Time						
	E8	228.87	497.31^a^	465.97^a^	88.36	92.34
	E13	239.22	487.65^a^	470.26^a^	94.78	93.58
	E18	212.42	450.55^b^	428.89^b^	88.81	85.15
	E23	222.96	437.29^b^	413.59^b^	89.77	87.65
SEM		8.33	14.06	13.73	3.66	4.54
Dose						
	0	224.01	471.65	452.35	87.89	87.68
	2	225.93	468.61	440.78	93.27	87.74
	4	214.92	455.75	425.02	85.22	88.24
	8	238.61	476.79	460.57	95.34	95.05
SEM		8.54	14.41	14.07	3.75	4.65
*P*-value						
Time		0.207	0.012	0.013	0.677	0.525
Dose		0.269	0.729	0.273	0.230	0.537
Time*dose		0.487	0.512	0.973	0.880	0.953

SEM, Standard error of the mean.

^a−b^Values within a column with no common letters differ significantly (P < 0.05).

^1^Each value represents the mean of 6 replicates.

**Table 4:** Effect of time and doses of an embryo injected OTA on the jejunal morphology of ducks at hatching^1^.

**Item**		**Villus height (μm)**	**Crypt depth (μm)**	**V/C**
**Time**	**Dose**			
E8	0	264.66	90.06	3.03
E8	2	184.36	78.61	2.38
E8	4	270.86	71.71	3.76
E8	8	232.84	76.47	2.88
E13	0	178.81	66.62	2.86
E13	2	227.44	77.60	2.77
E13	4	193.78	75.02	2.60
E13	8	169.79	68.71	2.19
E18	0	225.14	75.68	3.18
E18	2	161.14	81.19	1.98
E18	4	147.00	87.92	1.67
E18	8	230.17	80.19	2.88
E23	0	207.47	75.37	2.74
E23	2	199.49	79.51	2.52
E23	4	178.59	79.86	2.31
E23	8	184.55	76.50	2.43
SEM		57.191	10.282	0.707
*P*-value		0.371	0.246	0.161
Main effect				
Time				
	E8	238.18	79.21	3.01
	E13	192.45	71.99	2.60
	E18	190.86	81.24	2.43
	E23	192.52	77.81	2.50
SEM		14.178	2.370	0.175
Dose				
	0	219.02	76.93	2.95
	2	193.11	79.23	2.41
	4	197.55	78.63	2.58
	8	204.34	75.47	2.59
SEM		16.715	2.275	0.190
*P*-value				
Time		0.496	0.080	0.585
Dose		0.297	0.493	0.103
Time*dose		0.339	0.400	0.180

SEM, Standard error of the mean.

^1^Each value represents the mean of 6 replicates.

V/C: Ratio of Villus height to Crypt depth.

### Jejunal antioxidant capacity

The effect of time and doses of an embryo injected OTA on jejunal antioxidant capacity of ducks at hatching was shown in Table 5. There was no interactive effect between OTA injected time and doses on the jejunal antioxidant capacity, namely, SOD activity, T-AOC activity, and MDA content (*P* > 0.05). OTA injected time and doses influenced jejunal SOD activity (*P* < 0.05). OTA injected time had an effect on jejunal T-AOC activity and MDA content (*P* < 0.05). Regardless of OTA injected doses, the activity of SOD and T-AOC activities in the jejunum of ducklings injected with OTA at the 8^th^ and 13^th^ embryonic age was lower than that injected at the 18^th^ embryonic age (*P* < 0.05). The highest MDA content was observed in ducklings injected with OTA at the 13^th^ (*P* < 0.05).

**Table 5:** Effect of time and doses of an embryo injected OTA on the jejunal antioxidant capacity of ducks at hatching^1^.

**Item**		**SOD (U/mgprot)**	**AOC (mmol/g)**	**MDA** **(nmol/ mgprotein)**
**Time**	**Dose**			
E8	0	3.62^e^	0.101^c^	3.57^bc^
E8	2	4.53^de^	0.185^abc^	2.94^c^
E8	4	4.08^de^	0.151^bc^	2.67^c^
E8	8	4.71^de^	0.141^bc^	2.42^c^
E13	0	6.22^de^	0.201^ab^	5.14^ab^
E13	2	5.46^de^	0.091^d^	5.01^ab^
E13	4	4.38^de^	0.142^bc^	5.94^a^
E13	8	4.67^de^	0.097^bc^	4.72^a^
E18	0	7.34^bcd^	0.227^a^	2.45^c^
E18	2	11.48^a^	0.155^bc^	4.64^ab^
E18	4	9.32^ab^	0.198^ab^	2.30^c^
E18	8	8.70^bc^	0.237^a^	3.70^bc^
E23	0	6.61^cd^	0.188^ab^	4.19^a^
E23	2	8.88^bc^	0.176^abc^	4.12^abc^
E23	4	9.48^ab^	0.152^abc^	6.17^a^
E23	8	7.96^bcd^	0.107^bc^	4.47^a^
SEM		1.971	0.0462	1.508
*P*-value		<0.0001	0.0377	0.0006
Main effect				
Time				
	E8	4.23^b^	0.145^b^	2.90^b^
	E13	5.18^b^	0.133^b^	5.20^a^
	E18	9.21^a^	0.204^a^	3.27^b^
	E23	8.23^a^	0.156^b^	4.74^a^
SEM		0.454	0.0176	0.347
Dose				
	0	5.95^b^	0.179	3.84
	2	7.59^a^	0.152	4.18
	4	6.82^ab^	0.161	4.27
	8	6.51^b^	0.145	3.83
SEM		0.467	0.0176	0.36
*P-*value				
Time		<0.0001	0.014	<0.0001
Dose		0.042	0.4513	0.665
Time*dose		0.1327	0.0816	0.134

SEM, Standard error of the mean.

^a−b−c−d−e^Values within a column with no common letters differ significantly (P < 0.05).

^1^Each value represents the mean of 6 replicates.

SOD, Superoxide Dismutase; T-AOC, Total antioxidant capacity; MDA, Malondialdehyde.

### Jejunal inflammation cytokines

The OTA-injected time, doses, and their interaction had no effect on jejunum TNF-α level (Table 6, *P* > 0.05). OTA injected time, doses, and their interaction had an effect on jejunum IL-1β level (*P* < 0.05). Regardless of OTA injected doses, the jejunum IL-1β level of ducklings injected with OTA at the 13^th^ embryonic age was higher than other groups (*P* < 0.05). The jejunum IL-6 level of ducklings injected with OTA at 8^th^ and 13^th^ embryonic age was lower than at other injected ages. Regardless of OTA injected time, the embryo injected with OTA had a higher IL-1β levels than the control group (*P* < 0.05).

**Table 6:** Effect of time and doses of an embryo injected OTA on the jejunal inflammation cytokines of ducks at hatching^1^ (ng/gprotein).

**Item**		**IL-1β**	**IL-6**	**TNF-α**
**Time**	**Dose**			
E8	0	2.321^c^	0.433^c^	6.288
E8	2	1.914^c^	0.482^c^	7.547
E8	4	3.758^c^	0.448^c^	6.426
E8	8	4.460^c^	0.474^c^	13.589
E13	0	7.192^bc^	0.619^abc^	10.253
E13	2	19.419^a^	0.536^c^	11.542
E13	4	13.365^b^	0.436^c^	17.979
E13	8	13.258^b^	0.490^c^	16.560
E18	0	7.290^bc^	0.796^a^	12.237
E18	2	4.460^c^	0.690^abc^	15.861
E18	4	1.866^c^	0.553^b^	6.845
E18	8	3.277^c^	0.579^b^	5.754
E23	0	2.072^c^	0.569^b^	7.187
E23	2	1.414^c^	0.666^abc^	12.099
E23	4	10.194^b^	0.754^ab^	19.071
E23	8	20.994^a^	0.591^bc^	13.266
SEM		4.735	0.152	7.255
*P*-value		<0.0001	0.003	0.187
Main effect				
Time				
	E8	3.114^c^	0.459^b^	8.462
	E13	13.308^a^	0.520^b^	14.083
	E18	4.224^c^	0.654^a^	10.174
	E23	8.669^b^	0.645^a^	12.906
SEM		1.2788	0.0350	1.7985
Dose				
	0	4.719^b^	0.604	8.991
	2	6.802^a^	0.594	11.763
	4	7.296^a^	0.548	12.580
	8	10.498^a^	0.534	12.292
SEM		1.324	0.035	1.873
*P-*value				
Time		<0.0001	0.0002	0.269
Dose		0.0163	0.2992	0.540
Time*dose	0.0005	0.1784	0.132	

SEM, Standard error of the mean.

^a−b−c^Values within a column with no common letters differ significantly (P < 0.05).

^1^Each value represents the mean of 6 replicates.

## Discussion

### Hatching weight and organ index

Body weights of ducks hatched from OTA-contaminated eggs were significantly lower as compared with the control group. This decrease in the body weight is attributed to the adverse effects of OTA, particularly, a decrease in protein synthesis vital for the development of embryos (18). However, no such signs were observed in the present study. The adverse effects of OTA were not shown to lead to the hatching weight having a significant change on different OTA doses. The injection time significantly influences the hatching weight and the relative weight of the spleen of ducks. The reason for this result may be that attributed to the toxicity of OTA, particularly influencing the development of embryos. Early developmental stages in developing duckling embryos were more sensitive to OTA. We hypothesized that the spleen developed rapidly at the embryonic age of 18, and the immune function was basically complete. Low-concentration OTA stimulation promoted the immune response.

### Intestinal index

A study reported that the primary site of OTA absorption is the small intestine, and the site of maximal absorption is the jejunum compared with the other organs (19). The relative weight and length reflect the level of the growth and development of the intestine. The relative length of jejunum and ileum was significantly decreased on E18 and E23. The reason for these results was attributed to the enterotoxicity of OTA which made the intestine lighter and shorter. This needs to be further studied.

Intestinal villus height, crypt depth, and Vh/Cd are important indexes to measure intestinal digestion and absorption capacity. In this experiment, the toxicity of OTA was not expressed in modifications of intestinal morphology, namely, Villus height and Crypt depth on different injection times and OTA doses. No information appears to be available in the literature regarding the effect of embryo exposure OTA of ducks on villus morphology. But some studies reported that the cytotoxicity of OTA was expressed in modifications of villus morphology, consisting of shortened villi, hyperplastic crypts, and the appearance of prismatic epithelium with altered brush border (5). The enterotoxicity of OTA was observed in the terms of deleterious modifications in villus architecture, grossly evident in the shortened villi and elongated crypts (5, 20). For the results of this study, we speculated that no significant difference in villi morphology was because of the short time of embryo exposure to OTA. The intestine develops rapidly in the late stage of incubation (21). The concentration of absorbed OTA may be low due to the weak intestinal absorption capacity in the early stage of incubation.

### Jejunal antioxidant capacity

The OTA causes oxidative injury in animals, thus severely affecting animal health and production performance. The antioxidant enzymes catalase (CAT) and SOD are considered the first line of cellular defense against oxidative damage (22). MDA is one of several low-molecular weight end products formed *via* the decomposition of certain primary and secondary lipid peroxidation products (23). MDA production alters membrane fluidity and increases membrane fragility (24). Aflatoxin B1, OTA, and zearalenone combined with goat milk have been reported to significantly increase serum MDA, while SOD, and T-AOC levels decreased significantly (25). Dietary OTA decreased the antioxidant capacity of the intestine and increased the content of MDA (26, 27). No information appears to be available in the literature regarding the effect of embryos exposed to OTA for the intestinal antioxidant activity. In our study, the activity of SOD significantly decreased with the OTA dose increased leading to the antioxidant activity decreased. This was in agreement with a study (25) reported. The activity of SOD significantly increased on E18 and E23 may be due to the intestine being basically fully developed at the late stage of embryonic development, and low-concentration OTA stimulates the intestinal barrier to produce the antioxidant reaction. The relative length of jejunum on E13 was longer than on E18 and E23. The OTA exposed in the intestine had a larger contact area and absorbed more OTA, resulting in the higher content of MDA on injection time of E13. The results of this study showed that the embryo exposed to OTA was also accompanied by oxidative stress in the intestine of the duck at hatching. It also reduced the intestinal antioxidant capacity and increase the content of MDA.

### Jejunal inflammation cytokines

The enteric toxicity of OTA is mainly characterized by the oxidative stress and inflammatory response (28). High OTA concentrations damage the intestinal mucosa of broilers, leading to inflammatory effects that cause necrosis and shedding of the intestinal mucosa (29). The OTA is a fungal metabolite with immunomodulatory effects. A study has suggested that OTA induces the secretion of proinflammatory cytokines, such as IL-1β and tumor necrosis factor α, in the jejunum of Pekin ducklings (30). In addition, the intestinal barrier dysfunction is associated with intestinal inflammation, which is based on the destruction of tight junctions (31). Such inflammatory activity could result from a direct stimulatory effect on the production of pro-inflammatory cytokines by IECs (32). Indeed, it has been shown that Deoxynivalenol (DON), OTA, and Patulin (PAT) can directly stimulate cytokine production by immune cells (33–35), suggesting a possible similar direct effect of those mycotoxins on cytokines/interleukins production by gut epithelial cells. In addition to such direct effects, mycotoxin-induced intestinal inflammation could theoretically result from indirect proinflammatory effects. Moreover, mycotoxins could also indirectly cause intestinal inflammation through the opening of the tight junctions allowing the entry of luminal antigens and bacteria that are normally restricted to the gut lumen by the intestinal barrier function (36). In our study, the embryo injected with OTA significantly increased the content of IL-1β which was higher than other groups on E13. This result may be due to embryos being highly sensitive to the toxicity of OTA and are most sensitive on E13. OTA has a pro-inflammatory effect, and the toxicity of OTA maybe change the intestinal barrier through direct or indirect action, leading to a significant increase in the content of IL-1β. In conclusion, embryos exposed to OTA caused inflammation in ducks at hatching, leading to an increase in inflammatory factors.

## Conclusion

This study demonstrated the influence of embryonic exposure to OTA by different injection times and OTA doses for hatching quality and the jejunal antioxidant capacity of ducks at hatching. Embryo injected with ochratoxins A affected the hatching weight, the relative length of jejunum and ileum, decreased the antioxidant capacity and increased the content of proinflammatory cytokine IL-1β of the jejunum.

## Data availability statement

The original contributions presented in the study are included in the article/supplementary material, further inquiries can be directed to the corresponding author.

## Ethics statement

The animal study was reviewed and approved by Animal Care and Use Committee of Yangtze University.

## Author contributions

SZ, JL, and XJ conceived and designed the study and finished the first draft. JL, XJ, XP, YYu, and YS executed the experiment and analyzed the tissue samples. JL, XJ, XP, YL, ZY, XY, and YYa analyzed the data. SZ finished the revision. All authors interpreted the data, critically revised the manuscript for important intellectual contents, and approved the final version.

## Funding

This study was supported by the National Natural Science Foundation of China (32002219).

## Conflict of interest

The authors declare that the research was conducted in the absence of any commercial or financial relationships that could be construed as a potential conflict of interest.

## Publisher's note

All claims expressed in this article are solely those of the authors and do not necessarily represent those of their affiliated organizations, or those of the publisher, the editors and the reviewers. Any product that may be evaluated in this article, or claim that may be made by its manufacturer, is not guaranteed or endorsed by the publisher.
